# Prospective Evaluation of the Quality of Life of Patients after Surgical Treatment of Rectal Cancer: A 12-Month Cohort Observation

**DOI:** 10.3390/jcm11195912

**Published:** 2022-10-07

**Authors:** Magdalena Tarkowska, Iwona Głowacka-Mrotek, Bartosz Skonieczny, Michał Jankowski, Tomasz Nowikiewicz, Marcin Jarzemski, Wojciech Zegarski, Piotr Jarzemski

**Affiliations:** 1 Department of Urology, Nicolaus Copernicus University in Toruń, Collegium Medicum in Bydgoszcz, 85-094 Bydgoszcz, Poland; 2 Department of Rehabilitation, Nicolaus Copernicus University in Toruń, Collegium Medicum in Bydgoszcz, 85-094 Bydgoszcz, Poland; 3 Department of Surgical Oncology, Nicolaus Copernicus University in Toruń, Collegium Medicum in Bydgoszcz, 85-094 Bydgoszcz, Poland

**Keywords:** colorectal cancer, quality of life, laparoscopy, conventional surgeries

## Abstract

This study constitutes a prospective, three-stage evaluation of quality of life among patients receiving surgical treatment for colorectal cancer depending on the type of surgery performed (open anterior resection, laparoscopic anterior resection, abdominoperineal resection, or Hartmann’s procedure). The study included 82 patients treated at the Surgical Oncology Outpatient Department of the Oncology Center in Bydgoszcz from June 2019 to August 2021. The study tools consisted of diagnostic surveys and analyses of medical records. The standardized study tools were the surveys EORTC QLQ-C30 and QLQ-CR29. In addition, a proprietary questionnaire was developed to collect demographic data. Quality of life was measured at three time-points: the day before the surgery and 6 and 12 months post-surgery. Statistically significant differences (*p* < 0.05) were observed in the domains of role functioning (III, *p* = 0.030), body image (II, *p* < 0.001; III, *p* < 0.001), sexual functioning (II, *p* = 0.037), buttocks/anal area/rectum pain (III, *p* = 0.031), and embarrassment (II, *p* = 0.022; III, *p* = 0.010). Statistically significant differences in the functional and symptom scale scores were also observed within each group at different stages of cancer treatment. As shown by our study, the quality of life of patients treated for colorectal cancer is determined not only by the operating technique but also by sociodemographic and clinical factors. The use of minimally invasive surgical techniques enables patients to return to their social roles more quickly and improves their self-assessment of body image.

## 1. Introduction

A diagnosis of cancer is associated with stress, increased emotional tension, and uncertainty about distant outcomes. All these factors are frequently related to a reduction in the quality of life related to biopsychosocial functioning. Patients face new adaptational challenges, which frequently involve a change in the perception of their own body, a deterioration in physical fitness, and impairments in functioning in social roles [1]. One of the malignancies that significantly affects mental health and is responsible for significant changes in the corporeal aspect is colorectal cancer [2].

Colorectal cancer is the second most common cause of death from cancer in both genders in Poland and worldwide [2,3]. It is generally considered to be linked to the Western lifestyle; for example, a trend of increasing morbidity, including in people under 50 years of age, is observed as a result of changing dietary habits [4]. The primary method of radical treatment is surgical resection, frequently accompanied by perioperative systemic treatment or radiotherapy [5,6]; this approach yields good oncological outcomes [7] and is currently associated with 5-year survival rates of 61.6% to 70.9% [8].

The symptoms most commonly reported by patients include abdominal pain, changes in bowel movement patterns, changes in stool consistency, rectal bleeding, unjustified weight loss, depressed mood, and overall fatigue [9,10]. Notably, some of the complaints may be due to the specific type of surgery used to manage the disease. The difficulties encountered in patients whose surgery involved the formation of an intestinal stoma (stoma care, skin lesions, or unfavorable body image) are different from those in whom the continuity of the gastrointestinal tract is preserved (defecation and urination disorders, sexual disorders, or urges) [11,12]. If the severity of these symptoms is not reduced to an acceptable level in a timely manner, the patient may become predisposed to the development of mental disorders such as anxiety or depression or to adverse effects from the distant outcomes of treatment, such as health-adjusted quality of life [10].

The goal of the cohort study was to prospectively evaluate the quality of life in patients treated for rectal cancer depending on the type of surgery performed.

## 2. Material and Methods

This prospective, three-stage, cohort observational study with a pretest–posttest design included 82 consecutive patients admitted to the Surgical Oncology Outpatient Department of the Oncology Center in Bydgoszcz for surgical treatment of colorectal cancer. The patients were divided into four study groups according to the type of surgery they underwent: 21 patients were qualified for abdominoperineal resection (APR), 13 were qualified for Hartmann’s procedure (HART), 27 for anterior resection (AR), and 21 for laparoscopic anterior resection (LAR). The type of surgery depended on the location of the lesion. In most distal lesions, APR was performed, while in cases of possible sphincter preservation with no option of sigmoid-rectal anastomosis, Hartmann’s procedure was introduced. For most proximal lesions, either laparoscopic or classic anterior resection was carried out by random selection. Every procedure was attended by a high-volume surgeon (>50 colorectal operations per year). The team performing the procedures included 10 surgeons. Anal sphincter function was not assessed before the procedure.

Patients with a higher locoregional tumor stage and a higher risk of recurrence received neoadjuvant therapy consisting of radiation therapy with or without chemotherapy. All surgical procedures were performed at least 12 weeks after the initiation of radiotherapy and at least 4 weeks after the completion of chemotherapy, and they concerned only patients with residual tumor. In cases of complete response, resection was not performed. Adjuvant treatment was provided on the basis of pathomorphological diagnosis and consisted of chemotherapy or radiation therapy. Adjuvant treatment was provided to patients with a higher locoregional stage for up to 6 months after the treatment.

The assessments were performed at 3 time-points: the first survey was administered on the day prior to the surgical procedure, while the second and third measurements were performed 6 and 12 months after the procedure, respectively. The study was approved by the Bioethics Committee at the Nicolaus Copernicus University in Toruń (No. 283/2019). Participation in the study was voluntary. Each participant was familiarized with the study design and gave their written consent to participate in the research project. They received in-depth information about the objective of the study, the study conditions, and their right to withdraw at any time without stating the cause and without any consequences.

To ensure the uniformity of the sample and to exclude uncontrolled variables that could impact the study outcomes, inclusion and exclusion criteria were specified for the study.

*Inclusion criteria:*

− voluntary written consent to participate in the study
− good overall health status when recruited (Eastern Cooperative Oncology Group (ECOG) score of 0–1)
− hospitalization at the Clinical Department of Oncological Surgery of the Franciszek Łukaszczyk Oncology Centre in Bydgoszcz at the time of recruitment
− elective surgery
− no concomitant distant metastases
− rectal cancer qualified to radical resection: abdominoperineal resection, anterior rectal resection (open or laparoscopic), or Hartmann’s procedure
− clinical stage I–III (pTNM/ypTNM)
− age over 18 years
− good overall condition (ASA I–III)

*Exclusion criteria:*

− intraoperative conversion of surgical procedure
− uncontrolled psychiatric disorders
− other severe conditions that increase the risk of perioperative complications (ASA IV)
− continuity of the digestive tract being restored in the course of follow-up
− stoma formed during the first stage of the study procedure
− distant metastases
− stoma before surgery
− other surgical procedures in the post-surgical follow-up period
− survival of less than 12 months

The study was conducted using the diagnostic survey method. This method refers to the statistical collection of facts and information about structural and functional phenomena and their dynamics. The collected and grouped information on a given phenomenon allows us to determine its range, scope, level, and intensity. This in turn allows for an assessment of its causes and sequelae. Questionnaires are usually used in this method. The research tools included standardized questionnaires developed by the European Organization for the Research and Treatment of Cancer—the Quality of Life Questionnaire for Cancer Patients Version 3.0 (QLQ-C30) and the QLQ-CR29—the latter being a module dedicated to evaluating the quality of life of patients diagnosed with colorectal cancer. In addition, in order to evaluate the sociodemographic data of the study group, a proprietary questionnaire was constructed containing questions on age, gender, educational background, area of residence, employment status, number of children, marital status, and socioeconomic status.

The EORTC QLQ-C30 is a validated tool for the self-assessment of quality of life in patients diagnosed with cancer, regardless of its location or stage of development. The questionnaire consists of five functional scales (assessing physical, social, and emotional functioning, social roles, and memory and concentration), three symptomatic scales (fatigue, pain, and nausea and vomiting), and the core scale for overall self-assessment of quality of life and health. The scale also contains six questions relating to symptoms such as loss of appetite, diarrhea, constipation, financial difficulties resulting from illness, dyspnea, and sleeplessness.

The QLQ-CR29 is dedicated to assessing symptoms in patients diagnosed with colorectal cancer. The module contains 29 questions about the health problems experienced by patients treated for colorectal cancer. Better scores in the functional scales of the QLQ-C30 and QLQ-CR29 mean better performance in a particular aspect; with regard to the symptom scales, the higher the score, the higher the severity of the problem and the lower the quality of life [13].

The patients’ medical records were analyzed to obtain relevant clinical data. The type of surgery, BMI, type of neoadjuvant treatment, type of adjuvant treatment, duration of hospital stay (between the day of surgery and the day of discharge), and TNM grade of the disease were collected for statistical analysis purposes.

**Figure 1:**
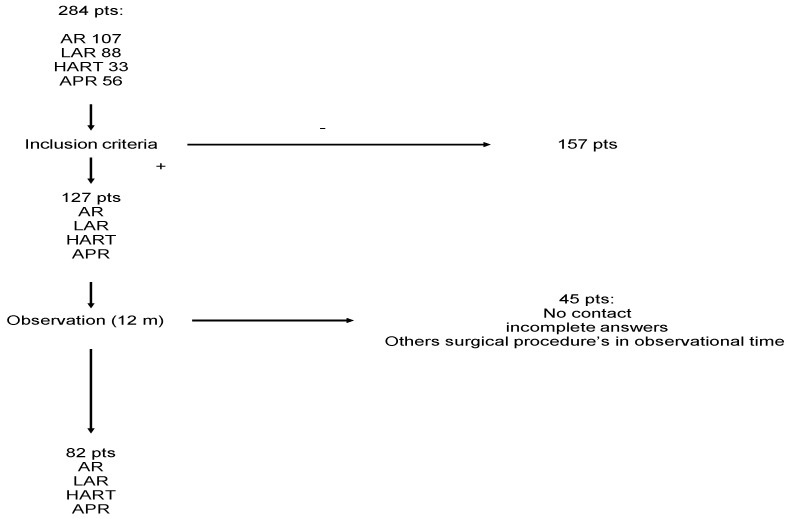
Diagram of patients excluded from the study.

At the first stage of the study (June 2019–August 2020), the qualification criteria were evaluated for a total of 107 anterior resections, 88 laparoscopic anterior resections, 33 Hartmann’s procedures, and 56 abdominoperineal resections performed within that period at the Clinical Department of Oncological Surgery of the Oncology Centre in Bydgoszcz, Poland.Due to the restrictions connected with COVID-19 in Poland, the first phase of study recruitment lasted from June 2019 to March 2020 and was followed by a two-month break until recruitment could continue in July and August 2020. A total of 127 patients meeting the inclusion criteria were included in the study. There were 45 patients excluded from the study due to gastrointestinal tract continuity being restored during the course of the follow-up, patients being lost to follow-up, or patients providing incomplete answers to survey questions. The final analysis was therefore carried out among the 82 patients who had met the inclusion criteria and completed all stages of the study. The details are presented in Figure 1.

## 3. Statistical Analysis

Statistical analysis was carried out using the software program IBM SPSS Statistics ver. 27.0 (IBM Corp. Released 2020. IBM SPSS Statistics for Windows, Version 27.0. IBM Corp., Armonk, NY, USA). Inter-group comparisons of demographic variables and medical data were carried out using Fisher’s exact test for nominal variables and a univariate ANOVA for quantitative variables. Inter-group comparisons of the quality of life at each of the three time-points were carried out using multivariate mixed-model ANOVA with the type of procedure and triple measurements corresponding to the inter- and intra-group variances, respectively. The homogeneity of variance in the groups was verified using Levene’s test. The sphericity of the covariance matrix was estimated using Mauchly’s test. If the sphericity assumption was violated, the Greenhouse–Geisser correction was applied. For multiple comparisons, the Bonferroni test was used with a correction for the significance level.

Demographic and medical data that significantly correlated with the overall health status were identified by means of multiple linear regression analysis using the enter method. The collinearity of predictors was controlled using the variance inflation factor (VIF). Heteroscedasticity was estimated using the Breusch–Pagan test. Autocorrelation in the residuals was estimated by the Durbin–Watson test and by the visual plot-based method. The normality of the distribution of the residuals was estimated from a histogram. Model predictors were selected on the basis of correlation analysis, with only those variables that were significantly related to the quality of life being included in the model. A probability value of *p* < 0.05 was used as the significance level and *p* < 0.01 was used as the level for highly significant differences.

## 4. Results

The study groups, as distinguished by the type of surgery (AR, LAR, APR, and HART), were characterized using demographic variables and clinical data. The groups did not differ in terms of demographic data: gender (*p* = 0.257), age (*p* = 0.511), educational background (*p* = 0.417), area of residence (*p* = 0.930), employment status (*p* = 0.577), number of children (*p* = 0.737), marital status (*p* = 0.088), and socioeconomic status (*p* = 0.872). The analysis of anthropometric data—body weight (*p* = 0.966), height (*p* = 0.879), and BMI (*p* = 0.690)—also revealed no statistically significant differences between groups.

The clinical characteristics of the study groups included the variables of adjuvant treatment (*p* = 0.491), duration of hospital stay (*p* = 0.204), and the TNM (tumor, nodes, metastases) grading of the disease (*p* = 0.204). No statistically significant differences were observed between the groups for these variables. As is shown by the analysis, the only significant difference between the groups was related to the type of neoadjuvant treatment (*p* = 0.021); however, the post hoc analysis failed to confirm such differences, which proved to be negligible (*p* > 0.05) in pairwise comparisons. It can therefore be assumed that the groups were similar in both demographic and medical terms. Male patients, urban residents, individuals in relationships, and those with two children prevailed in each of the groups. The average age of patients was about 62–67 years and the average BMI was about 25–27. The socioeconomic status of the respondents was reported as average or good in most cases. The mean length of hospital stay ranged between 7 and 13 days. The details are presented in Table 1.

Statistical analyses were carried out to compare the four patient groups, which were distinguished by the type of surgery, using the EORTC QLQ-C30 functional and symptom scale scores at three time-points. The functional scales taken into account in the analysis included overall quality of life (QL), physical functioning (PF), role functioning (RF), emotional functioning (EF), cognitive functioning (CF) and social functioning (SF). The higher the score, the higher the subject’s quality of life. At the first and second time-points, no significant differences were observed in any of the functional scale scores between the study groups (*p* > 0.05). At the third time-point, a statistically significant difference was observed in the role functioning scores (*p* = 0.030). A detailed post hoc analysis revealed that one year after the surgery, the quality of life related to role functioning was higher in patients who had undergone laparoscopic anterior resection as compared to those who had undergone open anterior resection of the rectum (*p* = 0.043). The differences between the other groups were not statistically significant (*p* > 0.05). For the remaining functional scales, no statistically significant differences were observed between the groups (*p* > 0.05).

The dimensions of the symptom scales that were taken into account were fatigue (FA), nausea and vomiting (NV), pain (PA), dyspnea (DA), sleeplessness (SL), loss of appetite (AP), constipation (CO), diarrhea (DI), and financial difficulties (FI). The higher the score, the greater the severity. The statistical analysis revealed no statistically significant differences between the groups (*p* > 0.05).

The next step consisted ofexamining the changes in quality of life within each group, as assessed using the EORTC QLQ-C30 functional and symptom scales at different time-points. In the group of patients classified for abdominoperineal resection, statistically significant differences were found in the scores corresponding to emotional functioning (*p* = 0.003), diarrhea (*p* = 0.024), and financial difficulties (*p* = 0.013). At time-point III, the emotional functioning level was much higher than at time-point I. The most severe symptoms on the diarrhea scale were observed prior to the surgery and they were completely resolved by time-points II and III. Financial difficulties were reported to be most aggravated 6 months after the procedure.

Among the patients classified for anterior resection of the rectum, statistically significant differences were found in the scores corresponding to social functioning (*p* = 0.004) and financial difficulties (*p* = 0.030). At time-point I, the quality of life in the social functioning domain was significantly higher than at time-points II (*p* = 0.014) and III (*p* = 0.006). No differences were observed between time-points II and III. Financial difficulties were also observed to be aggravated over time in this study group.

In the group of patients who qualified for LAR, significant differences were observed between the measurements within the emotional functioning domain. The reported level of social functioning at time-point II was significantly higher than at time-point I (*p* = 0.006), which means that a significant improvement had occurred within this domain.

**Figure 2:**
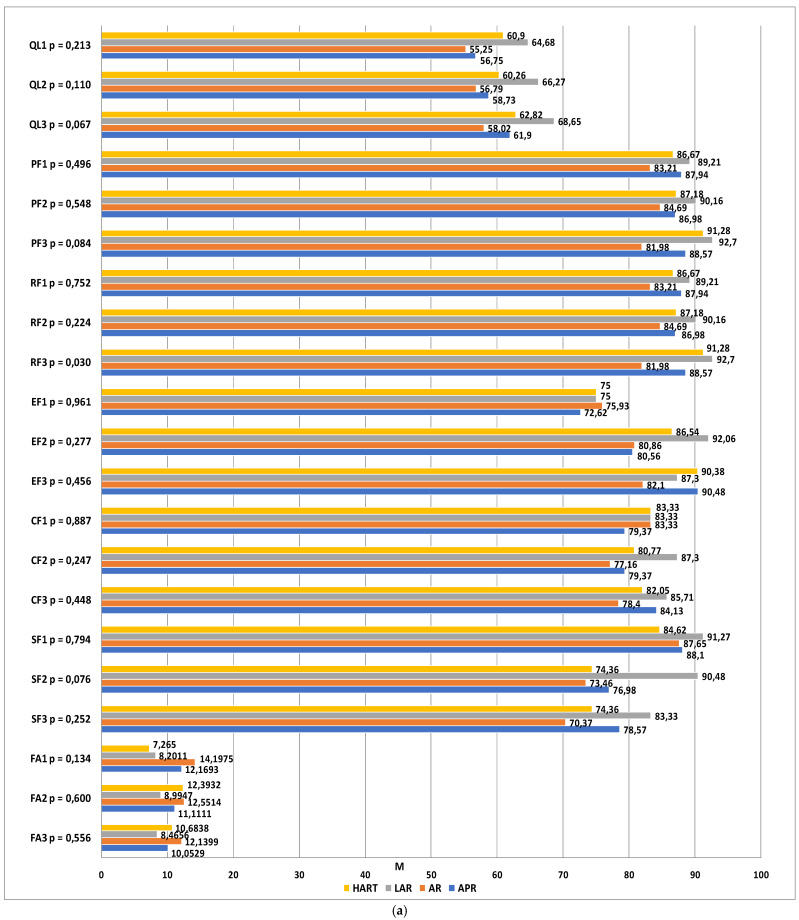

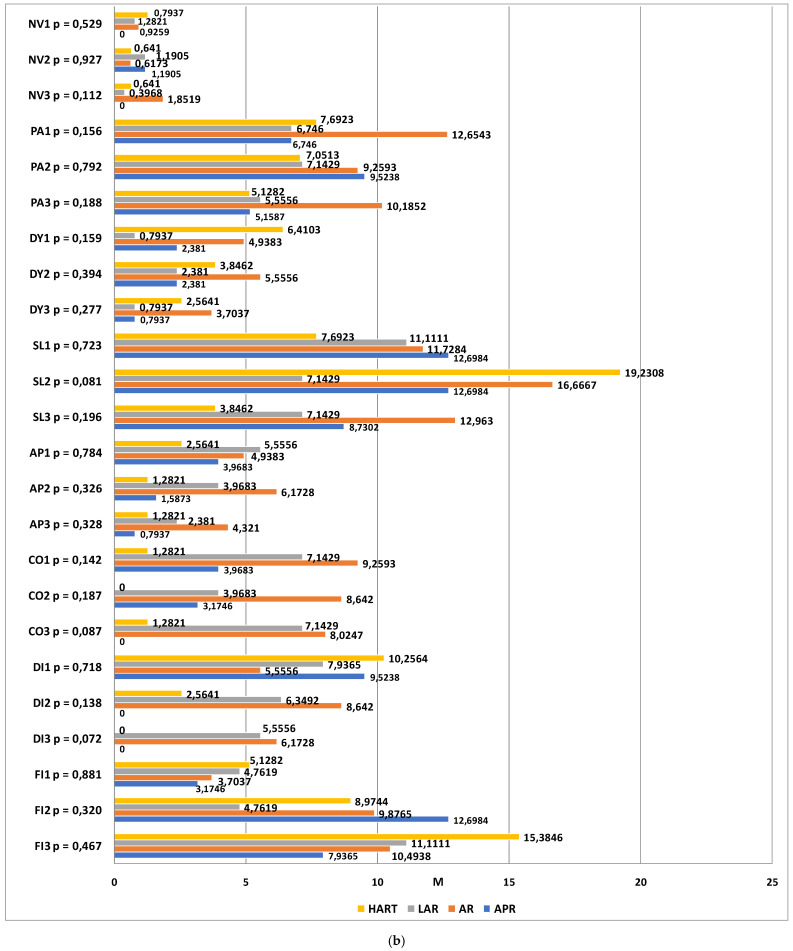
(**a**) Quality of life scores from the QLQ-C30 questionnaire showing the relationships between the groups (APR, AR, LAR, and HART) and the evaluation dates. QL—overall quality of life and health, PF—physical function, RF—role function, EF—emotional function, CF—cognitive function, SF—social function, FA—fatigue, *p*—significance level, M—mean. (**b**) Quality of life scores from the QLQ-C30 questionnaire showing the relationships between the groups (APR, AR, LAR, and HART) and the evaluation dates. NV—nausea and vomiting, PA—pain, DY—dyspnea, SL—sleeplessness, AP—loss of appetite, CO—constipation, DI—diarrhea, FI—financial difficulties, *p*—significance level, M—mean.

Among the patients who qualified for Hartmann’s procedure, significant differences were observed with regard to the sleeplessness scale (*p* = 0.004). Significantly more severe complaints were observed at time-point II as compared to time-points I (baseline) and III (one year after the procedure). Detailed results are shown in Figure 2a,b.

The QLQ-CR29 questionnaire included questions related to self-assessed body image (BI), anxiety regarding prospects in life (ANX), weight problems (WEI), sexual function in women (SEXW), and sexual function in men (SEXM). In addition, disease symptoms were assessed: urination frequency (UF), blood and mucus in stool (BMS), stool frequency (SF), urinary incontinence (UI), dysuria (DA), abdominal pain (AP), buttocks/anal area/rectum pain (BP), bloatedness (BF), dry mouth (DM), hair loss (HL), taste anomalies (TA), flatulence (FL), fecal incontinence (FI), skin sensitivity (SS), and embarrassment (EMB). The higher the score, the more severe the symptoms and the lower the functioning.

**Figure 3:**
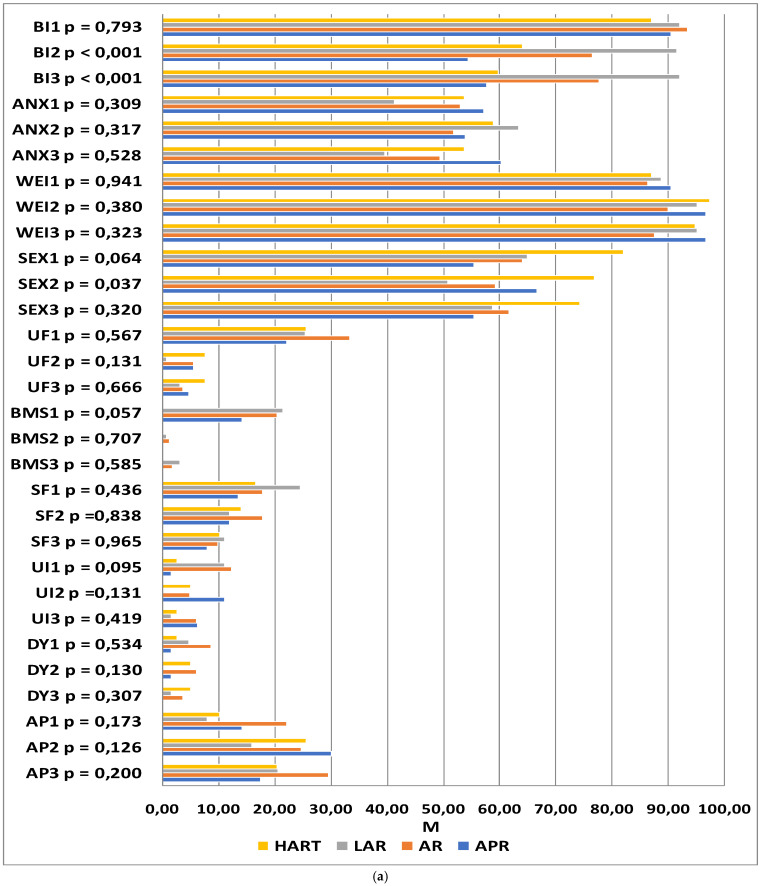

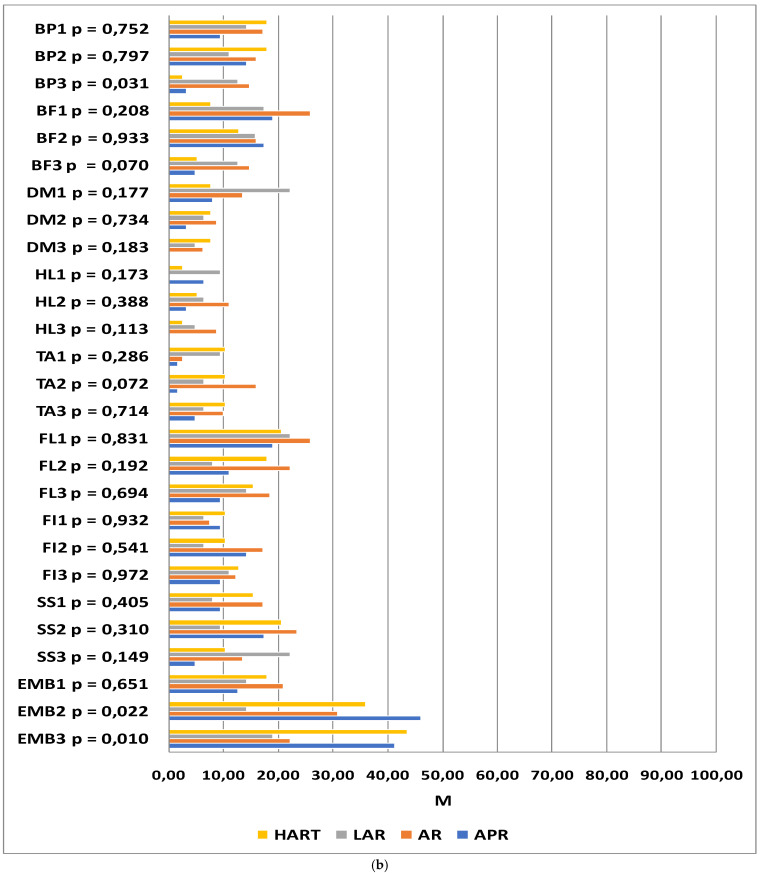
(**a**) Quality of life scores from the QLQ-CR29 questionnaire with the relationships between the groups (APR, AR, LAR, and HART) and the evaluation dates. BI—body image, ANX—anxiety regarding life perspectives, WEI—weight problems, SEX—sexual function in women, UF—urination frequency, BMS—blood and mucus in stool, SF—stool frequency, UI—urinary incontinence, DA—dysuria, AP—abdominal pain, *p*—significance level, M—mean. (**b**) Quality of life scores from the QLQ-CR29 questionnaire with the relationships between the groups (APR, AR, LAR, and HART) and the evaluation dates. BP—buttocks/anal area/rectum pain, BF –bloatedness, DM—dry mouth, HL—hair loss, TA—taste anomalies, FL—flatulence, FI—fecal incontinence, SS—skin sensitivity, EMB—embarrassment, *p*—significance level, M—mean.

At the first time-point, no significant differences were observed in any of the functional or symptom scale scores between the study groups (*p* > 0.05). At time-point II, statistically significant differences between the groups were reported with regard to body image (*p* < 0.001), sexual functioning (*p* = 0.033), and embarrassment (*p* = 0.022). The levels of embarrassment and difficulties accepting one’s own body image were significantly lower in patients who underwent laparoscopic anterior resection as compared to those who underwent abdominoperineal resection. The patients who underwent Hartmann’s procedure and abdominoperineal resection declared higher satisfaction with sexual functioning than those who underwent open or laparoscopic anterior resection. At time-point III, statistically significant differences between the groups were reported with regard to the scales of body image (*p* < 0.001), buttocks/anal area/rectum pain (*p* = 0.031), and embarrassment (*p* = 0.010). In the abdominoperineal resection patients, the difficulties accepting their body image were higher than in those who underwent laparoscopic anterior resection. Significant differences between the groups were also observed at time-point III on the scales of buttocks/anal area/rectum pain and embarrassment; however, the inter-group differences proved to be statistically insignificant after the significance level was corrected for multiple comparisons. The results of the analysis are summarized in Figure 3.

The next step consisted of analyzing the changes in quality of life within individual groups at successive time-points. In the patients who underwent abdominoperineal resection, statistically significant changes in quality of life were observed in the domains of body image (*p* = 0.001), urination frequency (*p* = 0.020), blood and mucus in feces (*p* = 0.025), abdominal pain (*p* = 0.006), buttocks/anal area/rectum pain (*p* = 0.049), bloatedness (*p* = 0.016), and embarrassment (*p* < 0.001). A highly significant deterioration in body image was observed at the second and third time-points as compared to the baseline. The severity of abdominal pain at time-point II was higher than that at time-points I and III. A simple effects test revealed that buttocks/anal area/rectum pain was more severe 6 months after the procedure than 12 months afterward. The incidence of bloatednesswas significantly lower at time-point III as compared to time-point II. The level of embarrassment at time-point I was significantly lower than at the other two time-points. The severity of urination frequency and blood and mucus in the stool at time-point I was significantly higher than at the two successive measurements, while the incidence of these symptoms at time-points II and III was at a similar level.

In patients having undergone AR, statistically significant changes in quality of life were observed in the domains of body image (*p* = 0.005), urination frequency (*p* < 0.001), blood and mucus in the stool (*p* < 0.001), hair loss (*p* = 0.028), and taste anomalies (*p* = 0.042). Significant deterioration was observed 6 and 12 months after the procedure in the domains of body image, hair loss, and taste anomalies as compared to the baseline measurements. The frequency of urination and the presence of blood and mucus in the stool were significantly higher at time-point I than the two successive measurements, though these symptoms were similar at time-points II and III.

LAR was associated with changes in quality of life as regards anxiety regarding prospects in life (*p* = 0.002), frequency of urination (*p* = 0.001), blood and mucus in the stool (*p* < 0.001), dry mouth (*p* = 0.008), and skin sensitivity (*p* = 0.038). With regard to anxiety, the highest scores were recorded 6 months after the procedure, with the results reported after 12 months being close to the baseline. The frequency of urination, the incidence of dry mouth, and the presence of blood and mucus in the stool were significantly higher at time-point I as compared to the two successive time-points, while these symptoms were similar at time-points II and III. With regard to skin sensitivity, the differences between the measurements were statistically significant; however, the significance disappeared after the results were adjusted for multiple comparisons.

In patients qualified for Hartmann’s procedure, statistically significant changes in the quality of life at successive time-points were observed in the domains of body image (*p* = 0.003), buttocks/anal area/rectum pain (*p* = 0.013), and embarrassment (*p* = 0.018). The lower scores in body image and the higher scores in embarrassment observed at time-points II and III were statistically significant. Buttocks/anal area/rectum pain was significantly lower 12 months after the procedure as compared to time-points I and II. The details are summarized in Figure 3a,b.

In order to determine which demographic variables and medical data were relevant as predictors of quality of life, a linear regression analysis was carried out using the enter method. The analysis revealed three statistically significant correlations with the explained variable at the first time-point: socioeconomic status, induction radiotherapy, and running one’s own business. The results suggest that quality of life is higher in individuals with higher socioeconomic status as well as in those running their own businesses instead of working on an employment contract. Induction radiotherapy was associated with a lower self-assessed quality of life.

The analysis for the second time-point indicated gender, marital status, and socioeconomic status as important predictors of quality of life. Higher quality of life was observed in male patients; additionally, the higher the socioeconomic status, the higher the quality of life. Singles reported lower quality of life scores than those in stable relationships.

At the third time-point, higher quality of life was measured in patients who had not received induction radiotherapy; likewise, quality of life was higher in those with higher socioeconomic status. The details are presented in Table 2.

The following section compares two groups of patients, those with and those without a stoma, using the functional and symptom scales of the QLQ-C30 questionnaire. The analysis revealed significant differences between these groups in the domains of constipation (time-point I: *p* = 0.032; time-point III: *p* = 0.011) and diarrhea (time-point II: *p* = 0.027; time-point III *p* = 0.008). Prior to surgery, the patients who qualified for treatment with preservation of gastrointestinal continuity were more likely to report constipation than patients who qualified for treatment with intestinal stoma formation. Similarly, one year after operation, the problem was reported more frequently by patients without a stoma. At time-points II and III, higher scores suggestive of higher symptom severity were reported for the diarrhea domain in the patients without an intestinal stoma.

Changes in quality of life, as reported at successive time-points, were observed between the stoma and no stoma groups. Statistically significant differences were found among the patients with intestinal stomas in role functioning (*p* = 0.031), emotional functioning (*p* < 0.001), social functioning (*p* = 0.041), sleeplessness (*p* = 0.013), diarrhea (*p* = 0.003), and financial difficulties (*p* = 0.005). In the group of patients with preservation of gastrointestinal continuity, significant differences in the scores reported at successive study time-points were reported for emotional functioning (*p* = 0.009), social functioning (*p* = 0.006), and financial difficulties (*p* = 0.014).

Among the patients in whom a stoma was formed during the course of the procedure, role functioning scores at time-point III were higher than at time-point II. Within the emotional domain, the quality of life of stoma patients was significantly lower at time-point I than at time-point III, whereas in patients in whom no stoma was formed in the course of the procedure, the quality of life at time-point I was significantly lower than at the two subsequenttime-points. In terms of social functioning, quality of life at time-point I was lower than at time-point II in stoma patients and lower than at time-point III in patients without a stoma. Increased financial difficulties were observed 6 and 12 months after treatment in both groups. The patients with stomas experienced a resolution of diarrhea problems, whereas the severity of sleeplessness increased by month 6.

The same analysis was performed with regard to the scales on the QLQ-CR29 questionnaire. It revealed significant intergroup differences within the domains of body image (time-point II: *p* < 0.001; time-point III: *p* < 0.001), sexual functioning (time-point II: *p* = 0.013), blood and mucus in the stool (time-point I: *p* = 0.029), urinary incontinence (time-point I: *p* = 0.012), buttocks/anal area/rectum pain (time-point III: *p* = 0.003), bloatedness (time-point III: *p* = 0.009), hair loss (time-point III, *p* = 0.033), and embarrassment (time-point II: *p* = 0.014; *p* < 0.001). Statistically significant differences were also observed at subsequent time-points. Among the patients with a stoma, these pertained to the domains of body image (*p* < 0.001), urination frequency (*p* = 0.002), abdominal pain (*p* = 0.001), buttocks/anal area/rectum pain (*p* = 0.001), bloatedness (*p* = 0.012), skin sensitivity (*p* = 0.049), and embarrassment (*p* < 0.001). For patients without a stoma, there were statistically significant differences in the domains of body image (*p* = 0.049), urination frequency (*p* < 0.001), blood and mucus in the stool (*p* < 0.001), stool frequency (*p* = 0.023), urinary incontinence (*p* = 0.023), and dry mouth (*p* = 0.007).

Lower scores for body image were observed in both groups, although they were much lower in patients who had a stoma formed during the course of the procedure. Significantly higher levels of embarrassment, skin problems, and abdominal pain were also observed after the surgical procedures in this group of patients. In addition, lower incidence rates of bloatedness, buttocks/anal area/rectum pain, and urination frequency were observed 6 and 12 months after the procedure.

**Figure 4:**
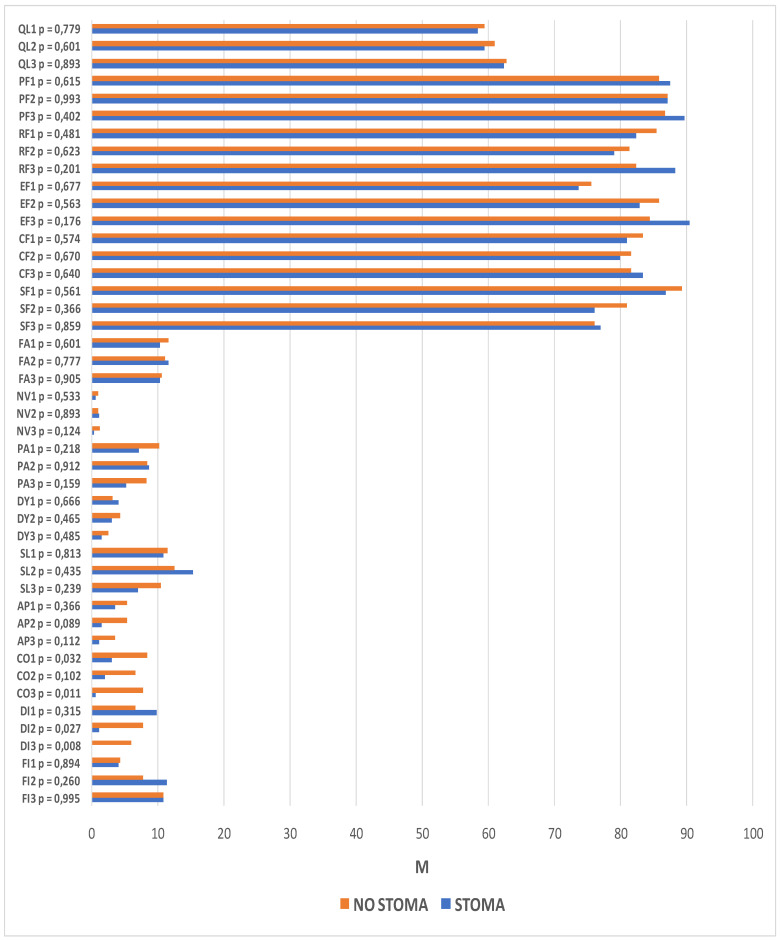
Quality of life scores from the QLQ-C30 questionnaire with the relationships between the groups (stoma/no stoma) and the evaluation dates. QL—overall quality of life and health, PF—physical function, RF—role function, EF—emotional function, CF—cognitive function, SF—social function, FA—fatigue, NV—nausea and vomiting, PA—pain, DY—dyspnea, SL—sleeplessness, AP—loss of appetite, CO—constipation, DI—diarrhea, FI—financial difficulties, *p*—significance level.

**Figure 5:**
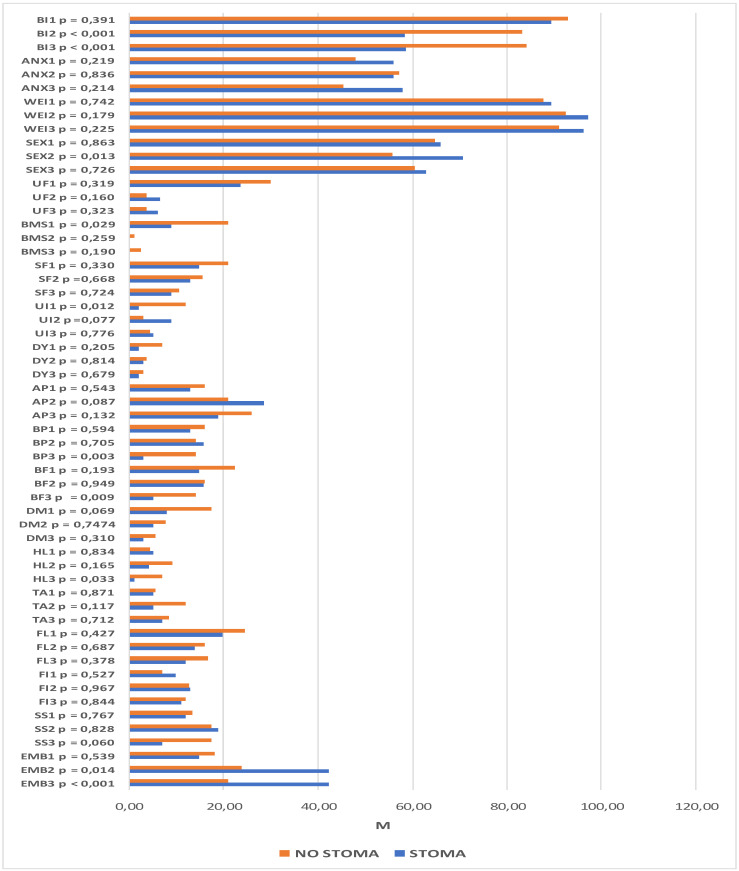
Quality of life scores from the QLQ-CR29 questionnaire with the relationships between the groups (stoma/no stoma) and the evaluation dates.BI—body image, ANX—anxiety regarding prospects in life, WEI—weight problems, SEX—sexual function in women, UF—urination frequency, BMS—blood and mucus in stool, SF—stool frequency, UI—urinary incontinence, DA—dysuria, AP abdominal pain, BP—buttocks/anal area/rectum pain, BF—bloatedness, DM—dry mouth, HL—hair loss, TA—taste anomalies, FL—flatulence, FI—fecal incontinence, SS—skin sensitivity, EMB—embarrassment, *p*—significance level, M—mean.

Significantly more buttocks/anal area/rectum pain, more frequent hair loss, and stronger bloatedness were reported 12 months after the procedure in patients in whom the continuity of the GI tract was preserved. In this group, there were lower incidence rates of blood and mucus in the stool, urinary incontinence, and both urination and stool frequency. More difficulties in sexual function were also observed 6 months after the surgery. The details are presented in Figure 4 and Figure 5.

## 5. Discussion

This paper assesses the quality of life of patients receiving surgical treatment for rectal cancer. Notably, the groups under analysis were not identical. The type of resection procedure is determined by the distance between the tumor and the anus. Distal tumors are more often associated with conditions such as rectal and pelvic pain, bleeding, defecation difficulties, and other symptoms that may significantly affect the patient’s quality of life prior to treatment. Similarly, the patient’s quality of life may be significantly affected by symptoms resulting from anterior resection and the associated intestinal anastomosis (constipation alternating with diarrhea or difficulties defecating), which depend on the distance between the anastomosis and the anus, among other factors; these are referred to as anterior resection syndrome (LARS). This syndrome is defined as bowel dysfunction leading to decreased quality of life [14]. Different groups of patients must be considered separately in order to understand the results of tests [14,15].

Numerous studies have confirmed the impact that patients’ perception of symptoms have on the distant outcomes of surgical treatment for colorectal cancer, including the quality of life component [1,2,16]. Our study shows that one year after surgery, the quality of life in terms of role functioning was higher in patients who underwent laparoscopic anterior resection than in those who underwent open anterior resection of the rectum. The results of other prospective studies describing the effect of colorectal cancer treatment on QLQ were [1,17]. Interestingly, as shown in our study, social role functioning in patients with a stoma 12 months after surgery had improved over the level measured 6 months after surgery and was even higher than before the surgery. In other prospective studies evaluating quality of life in patients with colostomy at different time-points after the surgery, the overall quality of life 6 months after surgery was found to be even better than in the pre-operative period, while no return to the baseline level has been observed for social role functioning, body image, chemotherapy side effects, or financial problems [18]. No significant differences were observed in this study in the global assessments of quality of life between the study groups or within individual groups at successive time-points. Similar data were provided by other authors, who likewise did not observe any links between the overall self-administered QLQ scores and the type of treatment provided [2,19]. Interestingly, no statistically significant differences were observed at any stage of our research with regard to the QLQ-C30 symptomatic scale scores as opposed to other authors [1].

Another objective of our research was to assess the changes in quality of life within each group in the months following the procedure. Better emotional functioning over baseline levels was observed in all study groups, the improvement being most evident in the patients who underwent laparoscopic anterior resection or abdominoperineal resection of the rectum. Other researchers have observed that after 12 months of surgery for colorectal cancer, emotional functioning was better than before surgery and even better than in the healthy population [17], while mental distress caused a significant QLQ impairment [20].

In addition, our study demonstrated that the degree of sleeplessness assessed 6 months after surgery was higher among patients who underwent HART as compared to the baseline and the 12-month measurements. Similar complaints were described by other authors inpatients undergoing conventional open surgeries [1]. Randomized studies suggest that moderate physical activity in these patients may relieve fatigue, insomnia, and pain [21]. In our study, the patients who underwent abdominoperineal resection were relieved from diarrhea-related problems, though they experienced more financial difficulties. Although financial problems were observed in all patients regardless of the type of procedure, they were least pronounced in the patients who had laparoscopic anterior resection. Financial liquidity is of particular importance in patients whose surgical procedure involved the formation of a stoma [22]. Other studies also highlighted financial issues as a factor that correlated with quality of life [23,24].A significant deterioration in the social functioning domain was observed over time in patients undergoing open anterior resection of the rectum. Other authors also take up the topic of decreased QLQ in this aspect [23,25].

In our study, the levels of embarrassment and difficulty accepting the changes to one’s body 6 and 12 months after the procedure were significantly lower in the laparoscopic anterior resection patients than in the abdominoperineal resection patients, which may be due to the extent of the latter procedure and its aftermath, including bodily mutilation. Moreover, body image was observed to deteriorate at successive time-points in all study groups, except for the patients who underwent laparoscopic anterior resection. In addition, in relation to the patients with stomas and those in whom gastrointestinal continuity had been preserved, difficulties accepting the new physical appearance after surgery were observed in both groups, being significantly worse among the former. Other authors also showed that the presence of a stoma negatively influences many QLQ domains [21,23,26,27]. The lessons learned by other researchers suggest that a timely return to health and a good quality of life is largely dependent on selecting a minimally invasive surgical technique [17]. In our study, the level of embarrassment was markedly higher in patients who underwent abdominoperineal resection and Hartmann’s procedure, which might have been due to the formation of an intestinal stoma, which is in accordance with the results of other authors [28]. Less acceptance of one’s body, greater problems with sexual functioning, and more severe symptoms within the urinary system were also reported by patients who underwent abdominoperineal resection according to Kang Sung-Bum et al. [19].

At the third time-point, statistically significant differences between groups were also found within the domains of buttocks/anal area/rectum pain; patients who underwent open or laparoscopic anterior resection reported more severe pain in these areas. Bloatedness, frequent urination, and the presence of blood and mucus within the feces were reduced after surgery regardless of the group. In the patients who underwent abdominoperineal resection, buttocks/anal area/rectum pain was found to be most severe 6 months after the procedure. Hartmann’s procedure was associated with a significant reduction in buttocks/anal area/rectum pain 12 months after the procedure. The results of studies by other authors show that the levels of pain (*p* = 0.0189) are significantly higher in patients who undergo anterior resection of the rectum [2], which contributes to QLQ impairment [24,29]. In our study, the self-assessed sexual function in patients treated with these methods was shown to be lower 6 months after the procedure. Heinsbergen et al. demonstrated the very negative impact of LARS on quality of life, including intimate life [30]. Other recent reports have confirmed that the type of incision and surgical technique have a significant impact on sexual dysfunction [1,31,32]. In our study, the patients who underwent laparoscopic resection presented with the lowest anxiety regarding prospects in life 6 months after the procedure; after 12 months, the assessments were close to those at the baseline, which is in accordance with the results of other authors [2].

The pre-treatment results obtained in our study suggest that the quality of life was higher in individuals with higher socioeconomic status and in those running their own businesses instead of working on an employment contract. Induction radiotherapy was associated with a lower self-assessed quality of life. Six months after surgery, a higher quality of life was reported by male patients, better-off patients, and singles. At the third time-point, a higher quality of life was measured in those patients who had not received induction radiotherapy; again, quality of life was higher in those with higher socioeconomic status. The relationship between demographic and clinical data quality of life was also confirmed in other studies [17,28,33,34,35,36,37,38].

In summary, these studies show that the quality of life of patients treated for colorectal cancer varies depending on the operating technique used, as well as sociodemographic and clinical factors. Despite its high scientific value, this study is burdened by several limitations. Firstly, the study groups were not randomized, and the patients’ eligibility was based on inclusion criteria alone. Secondly, the first measurement of quality of life was carried out the day before the procedure, which might have distorted the results in the emotional domains. The study groups were not identical in terms of perioperative treatment, and the patients in the LAR group were the least likely to have undergone preoperative treatment. No analysis of the potential correlations between quality of life and the distance between the tumor and the anal line was carried out within the groups of patients subjected to different operating techniques. It should also be noted that in most patients, the treatment started with induction radiotherapy, which, when used in the treatment regimen applied in the study patients, led to tumor downstaging, which could not have had no effect on their symptoms and QoL. Additionally, no analysis was carried out with regard to the adverse effects of neoadjuvant and adjuvant radiotherapy or chemotherapy. Moreover, it should be pointed out that no sphincter function was assessed before preoperative treatment or surgery. However, we believe that the symptoms of abnormal sphincter function were strictly dependent on the tumor stage and location, which determined the preoperative treatment and the type of surgery and thus naturally standardized the data. Moreover, no problems with stoma after APR or HART were observed. However, it should be pointed out that in both cases this is the same type of stoma, i.e., permanent end-sigmoid colostomy, which is connected with similar complications and sequelae.

It should also be underscored that it is impossible to directly compare all groups. However, the quality of life is in a direct correlation with factors determining the type of surgical treatment, e.g., tumor location. Only the LAR and AR groups may be compared to some extent. We did not refer to LARS directly in the analysis [15]. The syndrome could concern only patients subjected to AP/LAR and was not the subject of the analysis. On the other hand, the presence of LARS symptoms had a similar effect on QOL.

On the other hand, we believe that because this was a single-center study from a center with a high patient volume, the risks associated with the lack of standardized surgical procedures was reduced, which has a fundamental impact on the potential complications and outcomes of resection procedures.

This research is based on prospective studies, which reduces the risk of incorrect patient selection. This is a great advantage. However, the single-center character of the study determines another limitation, which is a small sample size.

## 6. Conclusions

Patients who underwent abdominoperineal resection or Hartmann’s procedure reported worse body image and greater embarrassment due to their condition than patients who underwent open or laparoscopic anterior resection of the rectum. However, sexual activity was improved; buttocks/anal area/rectum pain, bloatedness, and constipation were less severe; and hair loss was less common among these patients.

With regard to sociodemographic factors, the higher the economic status of patients, the higher their self-assessed quality of life. In addition, induction radiotherapy reduced the self-assessed quality of life. Significant deterioration in the economic status of subjects was observed, irrespective of the study group; this was least noticeable, however, among the patients who underwent laparoscopic anterior resection of the rectum.

As compared to open anterior resection of the rectum, laparoscopic anterior resection contributed to better role functioning one year after the procedure.

Anterior resection of the rectum was associated with a highly significant deterioration in the social functioning domain, which might have been due to anterior resection syndrome.

## Figures and Tables

**Table 1:** Comparison of demographic and medical data of patients in individual study groups.

	APR	AR	LAR	HART	*p*
Gender, *n (%)*					0.257
Female	8 (38.1)	12 (44.4)	10 (47.6)	2 (15.4)	
Male	13 (61.9)	15 (55.6)	11 (52.4)	11 (84.6)	
Age, *M (SD)*	62.76 (9.55)	65.04 (8.08)	63.24 (12.99)	67.61 (8.24)	0.511
Educational background, *n (%)*					0.417
Elementary	4 (19.0)	1 (3.7)	3 (14.3)	2 (15.4)	
Vocational	4 (19.0)	13 (48.1)	9 (42.9)	4 (30.8)	
Secondary	11 (52.4)	9 (33.3)	6 (28.6)	4 (30.8)	
Higher	2 (9.5)	4 (14.8)	3 (14.3)	3 (23.1)	
Area of residence, *n (%)*					0.930
Rural	7 (33.3)	8 (29.6)	6 (28.6)	5 (38.5)	
Urban	14 (66.7)	19 (70.4)	15 (71.4)	8 (61.5)	
Employment status, *n (%)*					0.577
Employed	3 (14.3)	5 (18.5)	6 (28.6)	5 (38.5)	
Housekeeping	1 (4.8)	2 (7.4)	0 (0)	0 (0)	
Retired	12 (57.1)	16 (59.3)	12 (57.1)	4 (30.8)	
Invalidity/family pension	3 (14.3)	3 (11.1)	2 (9.5)	4 (30.8)	
Self-employed	2 (9.5)	1 (3.7)	1 (4.8)	0 (0)	
Number of children, *n (%)*					0.737
0	0 (0)	3 (11.1)	1 (4.8)	1 (7.7)	
1	5 (23.8)	4 (14.8)	2 (9.5)	2 (15.4)	
2	11 (52.4)	13 (48.1)	12 (57.1)	9 (69.2)	
3	3 (14.3)	6 (22.2)	4 (19.0)	0 (0)	
4 or more	2 (9.5)	1 (3.7)	2 (9.5)	1 (7.7)	
Marital status, *n (%)*					0.088
In a relationship	17 (81.0)	21 (77.8)	17 (81.0)	6 (46.2)	
Single	4 (19.0)	6 (22.2)	4 (19.0)	7 (53.8)	
Socioeconomic status, *n (%)*					0.872
Low	1 (4.8)	2 (7.4)	0 (0)	0 (0)	
Average	7 (33.3)	14 (51.9)	9 (42.9)	7 (53.8)	
Good	12 (57.1)	10 (37.0)	11 (52.4)	6 (46.2)	
Very good	1 (4.8)	1 (3.7)	1 (4.8)	0 (0)	
Body weight, *n (%)*	75.14 (13.38)	77.33 (19.10)	77.10 (15.15)	75.92 (14.64)	0.966
Height, *n (%)*	169.52 (9.21)	167.07 (10.73)	167.69 (11.36)	167.65 (10.42)	0.879
BMI, *n (%)*	25.65 (5.54)	27.33 (4.43)	26.90 (3.70)	27.00 (5.09)	0.690
Neoadjuvant treatment, *n (%)*					0.021
None	1 (4.8)	9 (33.3)	8 (38.1)	0 (0)	
Chemotherapy	0 (0)	0 (0)	1 (4.8)	0 (0)	
Radiotherapy	8 (38.1)	6 (22.2)	6 (28.6)	6 (46.2)	
Radiochemotherapy	12 (57.1)	12 (44.4)	6 (28.6)	7 (53.8)	
Adjuvant treatment, *n (%)*					0.491
None	16 (76.2)	15 (55.6)	12 (57.1)	9 (69.2)	
Chemotherapy	5 (23.8)	12 (44.4)	8 (38.1)	4 (30.8)	
Radiotherapy	0 (0)	0 (0)	1 (4.8)	0 (0)	
Hospitalization days, *n (%)*	12.67 (13.02)	9.81 (7.16)	7.10 (4.21)	9.31 (5.69)	0.204
Disease stage (yp/pTNM), *n (%)*					0.577
I	12 (57.1)	5 (18.5)	8 (38.1)	5 (38.5)	
IIA	4 (19.0)	8 (29.6)	4 (19.0)	4 (30.8)	
IIB	0 (0)	1 (3.7)	0 (0)	0 (0)	
IIIA	1 (4.8)	2 (7.4)	0 (0)	0 (0)	
IIIB	2 (9.5)	7 (25.9)	6 (28.6)	3 (23.1)	
IIIC	2 (9.5)	4 (14.8)	3 (14.3)	1 (7.7)	

*n*—population, *p*—significance level, APR—abdominoperineal resection of rectum, AR—anterior resection of rectum, LAR—laparoscopic anterior resection of rectum, HART—Hartmann’s procedure.

**Table 2:** Coefficients of correlation between demographic and medical data and quality of life scores.

	Quality of Life—Time-Point 1		Quality of Life—Time-Point 2		Quality of Life—Time-Point 3
	*r*	*p*	*r*	*p*	*r*
Demographic data					
Gender	−0.13	0.236	−0.22	0.047	−0.15
Age	−0.01	0.980	0.01	0.907	0.07
Educational background	−0.15	0.190	−0.10	0.361	−0.09
Area of residence	0.08	0.480	−0.01	0.972	0.08
Employment status ^a^					
Housekeeping	−0.04	0.718	0.09	0.415	−0.02
Retired	0.02	0.843	0.07	0.522	0.01
Pension	−0.09	0.442	−0.04	0.738	−0.02
Self-employed	0.25	0.022	0.11	0.343	0.14
Number of children	−0.16	0.161	−0.06	0.642	−0.01
Marital status	−0.15	0.177	−0.22	0.048	−0.13
Socioeconomic status	0.23	0.037	0.23	0.041	0.22
Medical data					
BMI	0.09	0.410	0.10	0.348	0.09
Neoadjuvant treatment ^b^					
Radiotherapy	−0.23	0.041	−0.10	0.390	−0.27
Radiochemotherapy	0.07	0.554	−0.10	0.380	0.07
Adjuvant treatment ^b^					
Chemotherapy	0.06	0.601	0.04	0.693	−0.05
Hospitalization time	−0.15	0.167	−0.15	0.184	−0.20
Disease stage	−0.08	0.457	−0.10	0.363	−0.12

^a^—reference category: employed; ^b^—reference category: no treatment; r—correlation; *p*—significance level.

## Data Availability

The datasets generated and/or analyzed during the current study are available from the corresponding author upon reasonable request.
